# Effective Humanitarian Work: Teaching Medical Skill Sets in Ukraine

**DOI:** 10.5334/aogh.4306

**Published:** 2023-11-20

**Authors:** Michael S. Baker, Rom A. Stevens, Jacob Baker

**Affiliations:** 1 Osher LifeLong Learning, University of California, Berkeley, US; 2 Cal State University, East Bay, US; 3 Rosalind Franklin University of Medicine and Sciences, North Chicago, IL, US; 4 Merit Inc, US

**Keywords:** ATLS, StB, charitable donations, teaching medical skills

## Abstract

The senior authors traveled to Ukraine to teach specific skills to Ukrainian physicians and other medical professionals, utilizing a 2-day ATLS course, workshops in point-of-care ultrasonography (POCUS), lectures and webinars on damage control resuscitation, damage control surgery, and transfusion of whole blood. The authors have focused on providing skill sets that Ukrainian doctors can utilize within their existing system to improve immediate patient care for casualties resulting from the unanticipated Russian invasion and improve outcomes.

Given the resource limitations and differences of the Ukrainian healthcare systems, the authors believe Western-based professionals who come to Ukraine to help for short periods should resist the temptation to offer western solutions that may not work in Ukraine. Major improvements in Ukrainian health care will require long-term efforts in teaching but also need to include increased efforts to improve hospitals, clinics, staffing, education, supplies, and equipment.

Those who travel to help in Ukraine can still teach short courses that provide skills that Ukrainian doctors and nurses can use within their existing healthcare system to improve the quality of patient care in the immediate period of crisis and hopefully improve outcomes in the near term. It is not a reasonable expectation to think that the delivery of 2-day courses such as ATLS or POCUS will significantly change the country-wide delivery of healthcare. This sort of practice change requires the engagement of medical and political leaders and a sustained reform effort over years, not days or weeks. Supportive countries and non-governmental organizations need to prepare for a long and extensive investment in improving Ukrainian healthcare.

## Introduction

Dr. Michael Baker is a general and trauma surgeon working with the International Medical Corps (IMC) in conjunction with the Harvard Humanitarian Institute (HHI) to teach Advanced Trauma Life Support (ATLS) and Stop the Bleed (StB) in Ukraine. Dr. Rom Stevens is an anesthesiologist and critical care physician working with Help Ukrainian Hospitals, Inc., teaching point-of-care ultrasound (POCUS) workshops and lecturing on coagulopathy of trauma and whole blood transfusions in Ukraine. Both senior authors served in the U.S. Navy and Marine Corps, including combat tours. The information and opinions herein are based on their experiences in Ukraine during 2022–23.

Russia began its full-scale invasion of Ukraine on February 24, 2022. This act of unprovoked aggression is a violation of international law and the United Nations Charter and has been widely condemned by the international community. Health care providers, medical care delivery, hospitals, and clinics have been directly affected by Russian attacks [1] and indirectly through the loss of healthcare workers who became casualties, or who fled regions and cities under Russian occupation and/or attack. Targeting health care, although it violates the accepted laws of warfare, is being utilized by Russia to deliver tactical injury to the morale of the defenders [2].

Health system practice changes require a sustained training effort over years, the provision of resources, and the engagement of host nation leadership, which are difficult to accomplish in days or weeks. Many US-based non-governmental organizations (NGOs) have sent medical professionals to Ukraine. There are resource limitations and differences in the Ukrainian healthcare system that medical professionals from resource-rich countries may not appreciate during their short time in Ukraine. Hence, the authors have focused on providing skill sets that Ukrainian doctors can utilize within their existing system to improve immediate patient care and outcomes. In addition to teaching efforts, increased resources will be needed long-term to improve hospitals, clinics, staffing, education, supplies, and equipment.

## Medical Teaching in Ukraine

The Advanced Trauma Life Support^®^ (ATLS^®^) course is an example utilized to explain the authors’ thesis. ATLS teaches a concise approach to quickly assessing and managing injured patients. The American College of Surgeons Committee on Trauma (ACS CoT) has taught the ATLS course to over 1 million doctors in more than 80 countries. ATLS establishes a foundation of care for injured patients by teaching a common language and a common approach [3]. ATLS is also a component of U.S. military medical education, often taught to residents in training and frequently required or recommended by the US Department of Defense for many physicians, dentists, and physician assistants [4].

The ATLS course provides a standardized system for the evaluation and treatment of trauma victims. The course provides a paradigm for evaluation, treatment, education, and quality assurance and is a measurable, reproducible, and comprehensive system of trauma care.

ATLS didactic slides were translated into the Ukrainian language and checked by our interpreters for accuracy. A professional language interpreter was present for all didactic sessions and skill stations. Training videos had subtitles in Ukrainian along with interpretation of the lectures [5].

Multiple teams of western-based instructors provided two-day ATLS courses to Ukrainian physicians starting in August 2022, sponsored by IMC and HHI. Twenty cohorts of instructors have been deployed teaching in sites across Ukraine: Kyiv, Dnipro, Odessa, and Izmail. Instructors also taught a half day course called Stop the Bleed (StB) to medical personnel, support staff, administrative staff, and civilians during downtimes [6].

IMC reported that approximately 400 physicians completed ATLS and 1300 students completed Stop the Bleed over 15 months and 20 cycles of instructors (personal communication). A certificate of completion for ATLS and/or for Stop the Bleed was given to each student along with an individual first aid kit (IFAK), which contained a tourniquet, pressure dressings, and hemostatic gauze. The final ATLS class was extremely rewarding, as we had selected exceptional Ukrainian ATLS students and trained them as an instructor cadre who would teach the course going forward.

Attendees of these courses were attentive and engaged. They were especially grateful for this because of the wartime circumstances. A young Ukrainian physician reminded me after our first course, quoting from the Talmud, “…whoever saves one life saves the world entire [7].”

## Looking Back and Going Forward

The Ukrainian medical system is not comparable to a North American or western European healthcare system. It has mostly remained unchanged since gaining independence from the Soviet Union in 1991, although reforms were initiated by Dr. Ulana Suprun, Acting Minister of Health 2016–19 [8]. Since then, a change in government, the COVID-19 pandemic, and the war have impeded significant further reform.

The Ukrainian healthcare system is, in principle, free to the patient, with no charge for physician care, hospitalization, or medicines. However, free medications are limited to a Ministry of Health (MoH) list of medications. Any medications not on this list and not donated through humanitarian assistance must be purchased by the patient or family at a private pharmacy.

Ukrainian health care is underfunded (7% of GDP pre-war; EU average 10–11%, US > 18% GDP) with an excess capacity of hospital beds and hospitals. Physician and nurse salaries are low: anesthesiologist: 500 USD/month; resident: 125 USD/month [9]. Salaries are so inadequate that resident doctors often live with their parents to save money. Specialists may hold two or three jobs. Lack of adequate salaries encourages the persistence of “unofficial reimbursements,” whereby patients pay doctors money to ensure optimum care. Years of Soviet rule inculcated a mentality that patients and families must provide some form of reimbursement to the treating doctor to ensure good professional services [10].

The Ukrainian healthcare system, like many central European systems, is hierarchical. University professors, medical directors, and heads of departments have significant influence on clinical practice. The Ukrainian Ministry of Health (MoH) and Ministry of Defense (MoD) publish clinical practice guidelines, which are treated as directives to be followed without deviation by Ukrainian doctors. Instituting clinical practice changes in such a system will be a long process, involving specialty society leaders and officials within MoH as well as MoD.

Medical and nursing educational opportunities are limited by western-European standards. Specialty residencies are short (two years for internal medicine and family practice, three years for anesthesiology/critical care medicine, surgery, and orthopedic surgery). There are no subspecialty fellowships in the North American sense. Post-graduate medical education is very limited by western standards.

Nurses were in short supply in Ukraine before the war. Now nursing manpower is even more strained because some have been displaced from destroyed facilities or become refugees. There are fewer (than US) training opportunities, which limit nurses’ ability to engage in clinical decision-making. Limited numbers of nurses mean that some functions that would normally be performed in North America by nurses or therapists are performed by resident physicians, e.g., making ventilator adjustments, tube feedings, and mobilizing patients.

There is also a shortage of allied health professionals such as physical and occupational therapists, dieticians, etc. Given the large number of casualties, especially amputees, there are too few rehabilitation therapists (physiotherapy, occupational therapy). Mental health providers were in short supply before the war, and the demand signal now with millions of refugees, thousands of casualties, and the ongoing stress of war is difficult to meet with existing MoH and MoD resources. Experts state that 10 million people could be potentially at risk of mental disorders such as anxiety, depression, substance abuse, and post-traumatic stress disorder [11]. Currently, neither the MoD nor the MoH pay for outpatient mental health treatment. Out-of-pocket payment and support from charities are currently the only sources of funding for outpatient treatment of mental health disorders.

## Expectation of Medical Teaching in Ukraine

Adopting a western-style healthcare system at any time in the immediate future is not a reasonable expectation for Ukraine, due to a culture of universal free healthcare and limited national resources, now severely stretched by war. A major reform of the healthcare system in the middle of a fight for the survival of the nation is not likely. However, some things allies do can have significant benefits. In addition to teaching efforts, coordinating western-based charities to donate truly useful medical supplies and equipment can be helpful. Ukrainian hospitals currently rely heavily on donated pharmaceuticals and medical equipment.

Donations should be targeted to specific requests from specific hospitals to avoid random and unneeded medications or medical equipment that cannot be serviced in Ukraine due to a lack of a supply chain for specific spare parts or that is incompatible with the electrical and medical gas outlets. One example is a newly donated (June 2022) US-manufactured anesthesia machine purchased by a US-based charity for $50,000 USD. It now sits broken in a L’viv hospital, unable to be repaired due to a lack of spare parts and local expertise. Another example is the donation of 100 IV infusion pumps that were manufactured to US standards of 110V (Ukraine, like the rest of Europe, uses 220 V), and require a supple chain of disposable IV tubing, which is unaffordable in Ukraine. These items become “medical junk.” This waste of resources could have been avoided if western doctors with experience working in Ukraine had been engaged with NGOs and Ukrainian hospitals in decision-making.

Without language skills and knowledge of culture and history, visiting healthcare professionals will not have adequate context to provide useful help. We believe that western healthcare professionals who come to Ukraine to help must resist the temptation to offer western solutions that will not work and that will lead to frustration on the part of both parties.

**Figure 1:**
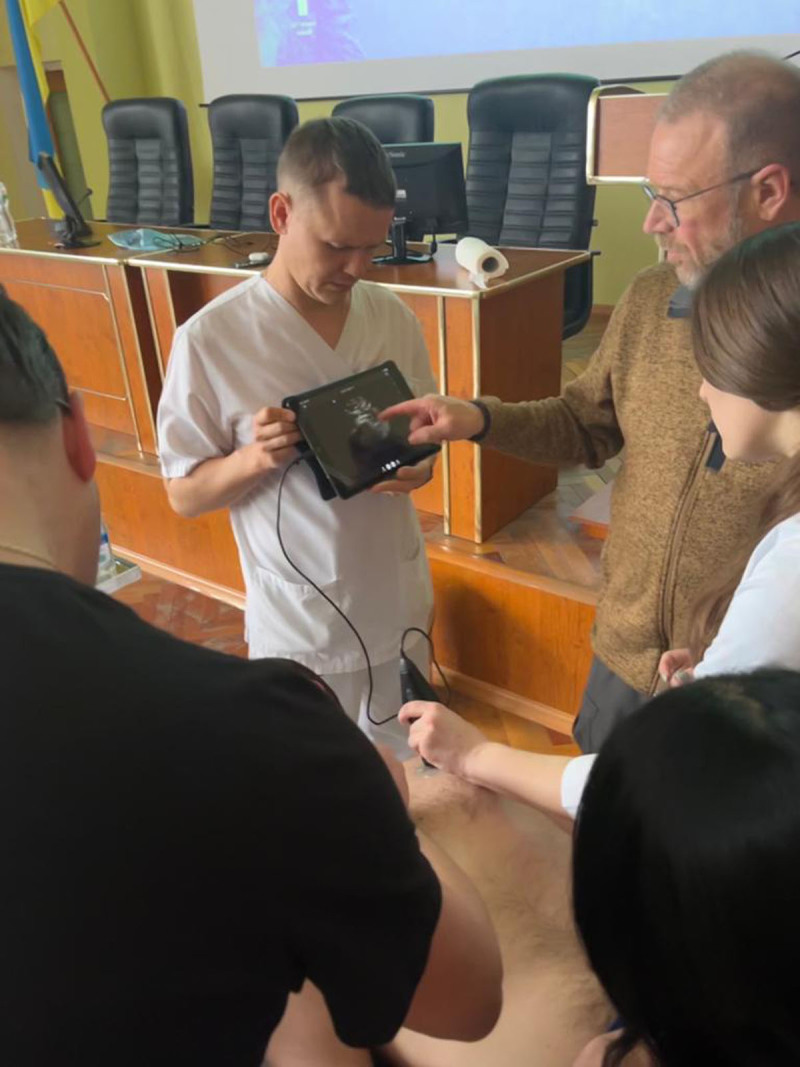
Dr Stevens – brown jacket-instructs student in ultrasound in Kalush City Hospital.

**Figure 2:**
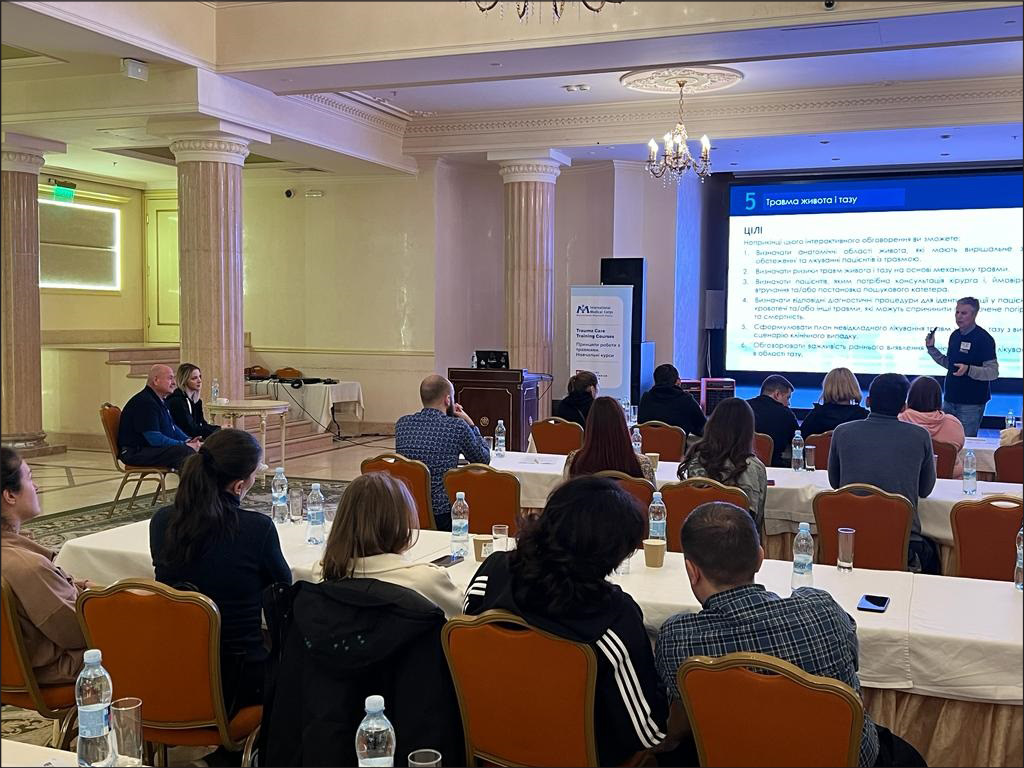
Dr Baker, far left with interpreter, certifying the instructor during ATLS training in Odesa March 2022.

That said, it is worthwhile to teach short courses to improve immediate patient care for traumatic injuries. Concentrating on providing specific skills, such as teaching a 2-day ATLS course or a one-day POCUS workshop (Figures 1 and 2), are examples that provide skills that Ukrainian doctors and nurses can integrate immediately into their skill set and utilize within their existing healthcare system to improve the quality of patient care in this period of crisis.

The front line of the fight for democracy in Europe runs through Kharkiv, Bakhmut, Zaporizhzhia, and Kherson. To provide effective support to the Ukrainian healthcare system, we must understand the limitations and risks. Long-term commitments by allied nations, charities, and partnerships with specific Ukrainian hospitals are needed to improve hospitals, clinics, staffing, education, equipment, and healthcare delivery. Ukrainians did not deserve this onslaught, and we must support the survival of their nascent democracy. “Slava Ukrayini” – Glory to Ukraine!
